# Phase I trial of oncolytic adenovirus-mediated cytotoxic and interleukin-12 gene therapy for the treatment of metastatic pancreatic cancer

**DOI:** 10.1016/j.omto.2020.11.006

**Published:** 2020-12-03

**Authors:** Kenneth N. Barton, Farzan Siddiqui, Robert Pompa, Svend O. Freytag, Gazala Khan, Irina Dobrosotskaya, Munther Ajlouni, Yingshu Zhang, Jingfang Cheng, Benjamin Movsas, David Kwon

**Affiliations:** 1 Department of Radiation Oncology, Henry Ford Cancer Institute, Henry Ford Health System, Detroit, MI 48202, USA; 2 Department of Gastroenterology, Henry Ford Cancer Institute, Henry Ford Health System, Detroit, MI 48202, USA; 3 Department of Oncology Hematology, Henry Ford Cancer Institute, Henry Ford Health System, Detroit, MI 48202, USA; 4 Division of Surgical Oncology, Department of Surgery, Henry Ford Cancer Institute, Henry Ford Health System, Detroit, MI 48202, USA

**Keywords:** clinical trials, pancreatic cancer, metastasis, gene therapy, immunomodulation, cytokines

## Abstract

The safety of oncolytic adenovirus-mediated suicide and interleukin-12 (*IL12*) gene therapy was evaluated in metastatic pancreatic cancer patients. In this phase I study, a replication-competent adenovirus (Ad5-yCD/*mut*TK_SR39_*rep*-hIL-12) expressing yCD/*mut*TK_SR39_ (yeast cytidine deaminase/mutant S39R HSV-1 thymidine kinase) and human IL-12 (*IL12*) was injected into tumors of 12 subjects with metastatic pancreatic cancer (T2N0M1-T4N1M1) at escalating doses (1 × 10^11^, 3 × 10^11^, or 1 × 10^12^ viral particles). Subjects received 5-fluorocytosine (5-FC) therapy for 7 days followed by chemotherapy (FOLFIRINOX or gemcitabine/albumin-bound paclitaxel) starting 21 days after adenovirus injection. The study endpoint was toxicity through day 21. Experimental endpoints included measurements of serum *IL12*, interferon gamma (IFNG), and CXCL10 to assess immune system activation. Peripheral blood mononuclear cells and proliferation markers were analyzed by flow cytometry. Twelve patients received Ad5-yCD/*mut*TK_SR39_*rep*-hIL-12 and oral 5-FC. Approximately 94% of the 121 adverse events observed were grade 1/2 requiring no medical intervention. Ad5-yCD/*mut*TK_SR39_*rep*-hIL-12 DNA was detected in the blood of two patients. Elevated serum *IL12*, IFNG, and CXCL10 levels were detected in 42%, 75%, and 92% of subjects, respectively. Analysis of immune cell populations indicated activation after Ad5-yCD/*mut*TK_SR39_*rep*-hIL-12 administration. The median survival of patients in the third cohort is 18.1 (range, 3.5–20.0) months. The study maximum tolerated dose (MTD) was not reached.

## Graphical Abstract

**Figure:**
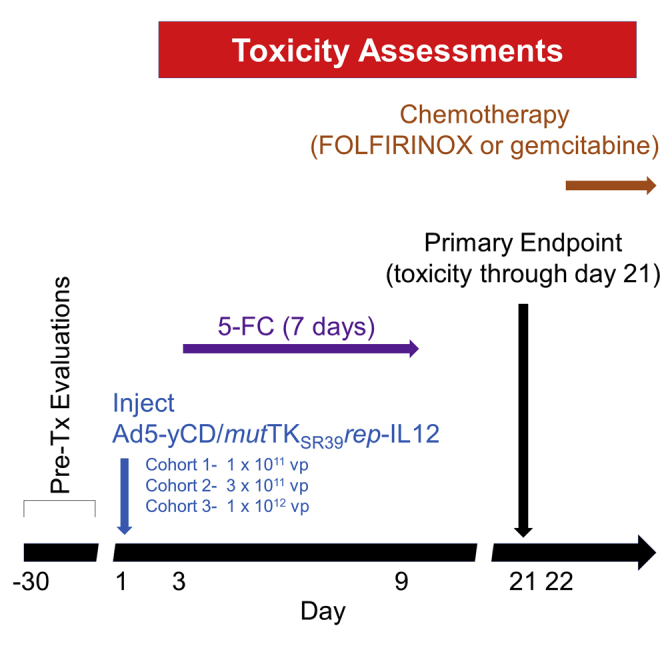

## Teaser

A phase I dose-escalation study was conducted to demonstrate the safety of oncolytic adenovirus-mediated suicide and interleukin-12 gene therapy combined with chemotherapy in metastatic pancreatic cancer patients. Our approach was shown to be safe, and there are indications of efficacy in patients who received the highest adenovirus dose.

## Introduction

According to the American Cancer Society, there will be an estimated 57,600 new cases of pancreatic cancer in 2020.^1^ Of those new cases, approximately 50% will be diagnosed as advanced or metastatic disease. In 2020, pancreatic cancer will become the second leading cause of cancer death, surpassing both breast and colorectal cancers. Optimal pancreatic cancer treatments depend on the stage at diagnosis and include a combination of surgical resection, chemotherapy, and/or radiation therapy. Even with the best treatments, the 5-year overall survival rate for all stages of pancreatic adenocarcinoma is 9.3% and a dismal 2.9% for metastatic pancreatic cancer patients.^2^ For locally advanced, unresectable, or metastatic pancreatic cancer, surgery is not always an option, and treatment goals include palliative therapy to prolong survival as best as possible. Standard treatment options for these patients include systemic therapy with and without radiation.^3^^,^^4^ For patients with good performance scores, FOLFIRINOX (leucovorin, 5-fluorouracil [5-FU], irinotecan, oxaliplatin) or gemcitabine + albumin-bound paclitaxel has become the standard of care. However, even with these first-line therapies, the overall survival of metastatic pancreatic cancer patients is 11.1 and 6.8 months for patients who received FOLFIRINOX and gemcitabine + albumin-bound paclitaxel, respectively.^5^ Toxicities associated with FOLFIRINOX limit its widespread application in patients with poor performance status. Owing to the limited survival duration of patients with metastatic pancreatic cancer, novel and alternative therapies are desperately needed.

Oncolytic adenovirus-mediated cytotoxic gene therapy is an approach developed and tested in several clinical studies. The oncolytic adenovirus delivers suicide genes to the target cells, which convert pharmaceutical agents into toxic metabolites locally. The suicide genes (yeast cytidine deaminase [yCD] and mutant S39R HSV-1 thymidine kinase [*mut*TK_SR39_]) have been evaluated in preclinical models and used in clinical studies for prostate cancer.^6^, ^7^, ^8^, ^9^, ^10^, ^11^, ^12^, ^13^, ^14^ Long-term survival data obtained in a previous clinical study suggested that the oncolytic adenovirus-mediated cytotoxic gene therapy triggered the immune system to target the tumor.^15^ We explored the concept of combining our suicide gene therapy with a cytokine to promote the immune system activation against tumors. An oncolytic adenovirus expressing the suicide genes and mouse interleukin-12 (mIL12) was constructed and tested in preclinical models.^16^ The injection of the newly constructed adenovirus into orthotopic tumors resulted in the following: (1) increased levels of serum mIL12 and interferon gamma (IFNG), and (2) increased numbers and activation of natural killer (NK) cells and cytotoxic T lymphocytes. Tumor control in these preclinical models was improved with the combined suicide gene therapy and *IL12* immunotherapy. Based upon these intriguing preclinical results, a new adenovirus was constructed expressing the human version of the *IL12* gene (Ad5-yCD/*mut*TK_SR39_*rep*-human IL-12 [*IL12*]) for testing in the clinic.

A phase I dose-escalation clinical trial was conducted to assess the safety of Ad5-yCD/*mut*TK_SR39_*rep*-hIL-12 in metastatic pancreatic cancer patients. These patients received a single injection directly into the pancreatic tumors of Ad5-yCD/*mut*TK_SR39_*rep*-hIL-12 at one of three escalating doses. Patients subsequently received standard-of-care chemotherapy at the discretion of the treating oncologist. As was done in previous clinical trials, the persistence of Ad5-yCD/*mut*TK_SR39_*rep*-hIL-12 DNA in the subjects’ blood was followed. We also evaluated whether we could detect, using flow cytometry, any activation of the immune system by assessing the number of peripheral blood mononuclear cells (PBMCs) and a subset of proliferation markers on those cells before and after treatment with Ad5-yCD/*mut*TK_SR39_*rep*-hIL-12. Similarly, we investigated the levels of serum *IL12*, INFG, and CXCL10 following Ad5-yCD/*mut*TK_SR39_*rep*-hIL-12 administration. Finally, we followed the survival of patients to assess the efficacy of the approach of combining oncolytic adenovirus-mediated cytotoxic gene therapy and *IL-12* cytokine therapy with chemotherapy in patients with metastatic pancreatic cancer.

## Results

### Patients and treatment

Between October 2017 and May 2019, 12 patients were enrolled (Table 1). The median age of the patients enrolled in the study was 68 (range 55–84) years. Of the patients enrolled in the study, nine were men and three were women; 10 patients were white and 2 African American. Eastern Cooperative Oncology Group (ECOG) performance scores ranged from 0 to 1. The median primary tumor dimensions were 3.3 cm (range, 2.3–5.9 cm) × 3.1 cm (2.0–4.3 cm) × 3.2 cm (1.5–5.1 cm). Tumor volumes ranged from 6 to 57 cubic centimeters (cc) with a mean value of 22 cc as determined by computed tomography (CT), magnetic resonance imaging (MRI), or endoscopic ultrasound (EUS). Extra pancreatic extension or lymph node involvement was observed in nine (75%) or seven (58%) of the patients, respectively. Encasement of major blood vessels was reported in four (25%) of the patients. Liver metastases were detected in all patients enrolled in the study. Other organs with potential metastases included kidney, lung, spleen, and adrenal gland.

**Table 1:** Clinical trial overview

	Subject											
	1	2	3	4	5	6	7	8	9	10	11	12
Age (years)	56	68	77	74	69	58	84	68	72	66	55	66
Gender	M	M	M	M	F	M	F	M	M	M	M	F
Race	W	AA	W	W	W	W	W	W	AA	W	W	W
ECOG PS	1	1	0	1	0	1	0	0	0	1	1	0
Location of primary tumor	B/N	B	H/N	H/U	T	H	B	H	T	B/T	B/T	T
Primary tumor size (cc)	10	25	23	19	8	18	8	6	57	17	53	22
Extrapancreatic extension	Y	Y	N	Y	Y	Y	N	N	Y	Y	Y	Y
Lymph node involvement	Y	Y	N	Y	N	Y	N	Y	N	Y	Y	N
CA/SMA encasement	N	Y	N	N	N	N	N	N	Y	Y	Y	N
Liver metastases	Y	Y	Y	Y	Y	Y	Y	Y	Y	Y	Y	Y
Tumor in other organs	N	N	kidney	lung	spleen	lung	N	N	Lu/Ki/Ad	lung	N	N
Tumor stage^a^	T2N1M1	T4N1M1	T2N0M1	T2N1M1	T2N0M1	T2N1M1	T2N0M1	T2N1M1	T4N0M1	T2N1M1	T4N1M1	T2N0M1
Cohort	1	1	1	2	2	2	3	3	3	3	3	3
Adenovirus dose (vp)	1 × 10^11^	1 × 10^11^	1 × 10^11^	3 × 10^11^	3 × 10^11^	3 × 10^11^	1 × 10^12^	1 × 10^12^	1 × 10^12^	1 × 10^12^	1 × 10^12^	1 × 10^12^
Chemotherapy regimen	FOL	G/A	none	G/A	FOL to G/A	FOL	G	G/A	FOLFIRI	FOL	FOL	FOLFIRI to FOLFOX to G/A
Status (as of 11/05/2020)	expired	expired	expired	expired	expired	expired	expired	alive	expired	alive	alive	alive
Number of grade >3 AEs^b^	1	1	0	3	0	1	1	0	0	0	0	0
Number of SAEs^c^	0	0	0	0	0	0	1 poss	0	0	0	0	0
Tumor response at 9 months	PD	PD	ND	SD	SD	PD	SD	SD	PD	SD	PD	PD
Survival (months)^d^	4.8	5.4	1.6	2.7	10.5	3.5	15.2	20.0	3.5	19.1	18.1	18.0
Adenovirus DNA in blood	N	N	N	N	Y	N	Y	N	N	N	N	N
IL-12/INF-g/IP10 in serum	N/Y/Y	N/Y/Y	N/N/N	Y/Y/Y	Y/N/Y	N/N/Y	Y/Y/Y	Y/Y/Y	Y/Y/Y	N/Y/Y	N/Y/Y	N/Y/Y

B, body; CA, celiac axis; CR, complete response; F, female; FOL, FOLFIRINOX; G, Gemzar; G/A, Gemzar/Abraxane; H, head; M, male; N, neck; ND, cannot be determined based on CT every 2–3 months; PD, progressive disease; PR, partial response; SD, stable disease; SMA, superior mesenteric artery; T, tail; U, uncinate process.

a American Joint Committee on Cancer, Eighth Edition.

b Grade ≥3 adverse events (AEs) through primary toxicity endpoint (day 21 after the adenovirus injection) and prior to the start of chemotherapy.

c Serious AEs (SAEs) that were unexpected and judged to be possibly/probably/definitely related to the investigational treatment (through day 21).

d At the time of manuscript preparation.

Median follow-up time of the surviving patients is 18.6 months. All patients enrolled in the trial received a single injection of adenovirus into the pancreatic tumor mass at the prescribed dose listed in Table 1. All patients received the prescribed dose of 5-fluorocytosine (5-FC) prodrugs according to protocol. Patients with good performance scores were offered FOLFIRINOX for as many cycles as they could tolerate. All but one patient in the current study received at least three cycles of chemotherapy at the discretion of the treating oncologist. Six patients in the current study started with FOLFIRINOX, while five received one or more other chemotherapy options. One subject (patient 3) expired after 1.6 months because of disease progression after the adenovirus was injected and before chemotherapy could be administered. The study design allowed for the endpoint to be reached (day 21) before chemotherapy would begin to avoid difficulty in the toxicity assessment.

### Toxicity

Over 94% of the adverse events (AEs) regardless of their attribution were mild (grade 1) or moderate (grade 2). The maximum tolerated dose (MTD) was not achieved. A possible severe AE (SAE) observed in one patient will be described in detail below. Treatment-related AEs could be attributed to Ad5-yCD/*mut*TK_SR39_*rep*-hIL-12 (chills, fatigue, flu-like symptoms, malaise, and increased serum alanine aminotransferase/serum glutamic-pyruvic transaminase [ALT/SGPT] and aspartate transferase/serum glutamic oxaloacetic transaminase [AST/SGOT]) or the 5-FC prodrug therapy (anemia, diarrhea, nausea, vomiting, leukopenia, lymphopenia, and thrombocytopenia) (Table S1). The incidence and severity of the AEs were nearly identical to those observed in five previous clinical studies employing three similar oncolytic adenoviruses.^9^, ^10^, ^11^, ^12^, ^13^, ^14^ A total of 121 AEs were recorded by the study endpoint (day 21). Of the recorded 121 AEs, there were six grade 3 (5.0%) and one grade 4 (0.8%) toxicities, including one report of hypotension, one episode of syncope, three incidents of hyperglycemia (including one grade 4), an account of lymphopenia, and one resultant spike in serum lipase. The reports of hypotension and syncope are related and will be described below. Hyperglycemia can be attributed to the underlying disease and comorbidities of the subjects. The grade 3 decrease in blood cell count is likely due to the 5-FC therapy and have been observed in previous clinical trials using similar agents.^9^, ^10^, ^11^, ^12^, ^13^, ^14^ The reduced blood count returned to normal levels within 72 h without intervention or modification of the study protocol.

The first subject (patient 7) in the third cohort, who received the highest dose of Ad5-yCD/*mut*TK_SR39_*rep*-hIL-12 (1 × 10^12^ viral particles [vp]), experienced nausea, vomiting, and dizziness after taking the first dose of 5-FC (Ancobon) medication on the morning of day 3 after adenovirus injection. Nausea and vomiting are common side effects of the Ancobon treatment. Upon standing up from a seated position, the subject fainted, fell, and hit the back of her head on the wall of the bathtub (grade 3 syncope). The subject was taken to the emergency department by members of her family. Her diastolic blood pressure (115/55 mm Hg) was below normal range (grade 3 hypotension). She was administered fluids intravenously, and the laceration on the back of her head was treated. The subject complained of headache but denied chest pains or shortness of breath. She had a slight fever (100.2°F). Otherwise, there were no significant abnormalities identified in blood chemistries. Resultant CT of the head and cervical spine showed no fractures or acute traumatic injury. Chest X-rays did not exhibit any etiology for her syncopal event. The subject was admitted to the hospital for observation and supportive care and was discharged the next day (day 4 from injection). She was seen twice in a multidisciplinary pancreas cancer clinic the following week (days 5 and 8), and all symptoms, except for nausea, fatigue, and lightheadedness in the morning, had resolved. The subject resumed treatment for her cancer on post-injection day 8. As per the study protocol, the SAE was reported to the study Data Safety Monitoring Board (DSMB) for review. In addition to informing the DSMB, the institutional review board (IRB) and US Food and Drug Administration (FDA) were also notified of the SAE. No patients were enrolled in the study during the DSMB review. One of the known side effects of *IL12* administration is hypotension mediated by IFNG induction.^17^ During the DSMB review, the SAE can be designated as being unrelated or possibly, probably, or definitely related to the study agent. The DSMB determined that the SAE was possibly related to the study agent in this case. In response to the DSMB assessment, the third cohort was expanded from three patients to six patients and accrual resumed. All five of the remaining patients in the highest cohort completed their treatments and reached the endpoint without encountering any SAE. Owing that only one of six patients (16%) in the highest dose cohort developed a dose-limiting toxicity (DLT) possibly related to the treatment, the study MTD was not reached.

### Efficacy

Although the study is designed to evaluate the safety of combining oncolytic adenovirus-mediated cytotoxic gene therapy and *IL12* cytokine therapy with chemotherapy in patients with metastatic pancreatic cancer, we also assessed the efficacy. The median survival of subjects who were administered 1 × 10^11^ or 3 × 10^11^ vp Ad5-yCD/*mut*TK_SR39_*rep*-hIL-12 had median survival durations of 4.8 (range, 1.6–5.4) and 3.5 (range, 2.7–10.5) months, respectively. All the patients who received these doses of Ad5-yCD/*mut*TK_SR39_*rep*-hIL-12 have expired. The two patients (patients 9 and 7) who received the highest dose of adenovirus expired at 3.5 and 15.2 months, respectively. At the time of writing this manuscript, four of the six patients who received the highest dose of Ad5-yCD/*mut*TK_SR39_*rep*-hIL-12 (1 × 10^12^ vp) are still alive, with a median survival duration of 18.1 (range, 3.5–20.0) months. As part of routine follow-up, patients undergo routine radiographic surveillance to assess disease progression. With a median CT imaging follow-up after adenovirus injection of 16.8 (range, 16.1–17.4) months, two of the four surviving patients were assessed to have stable disease in both the treated tumors (pancreas) and the metastases found at other sites.

### Experimental endpoints

**Figure 1:**
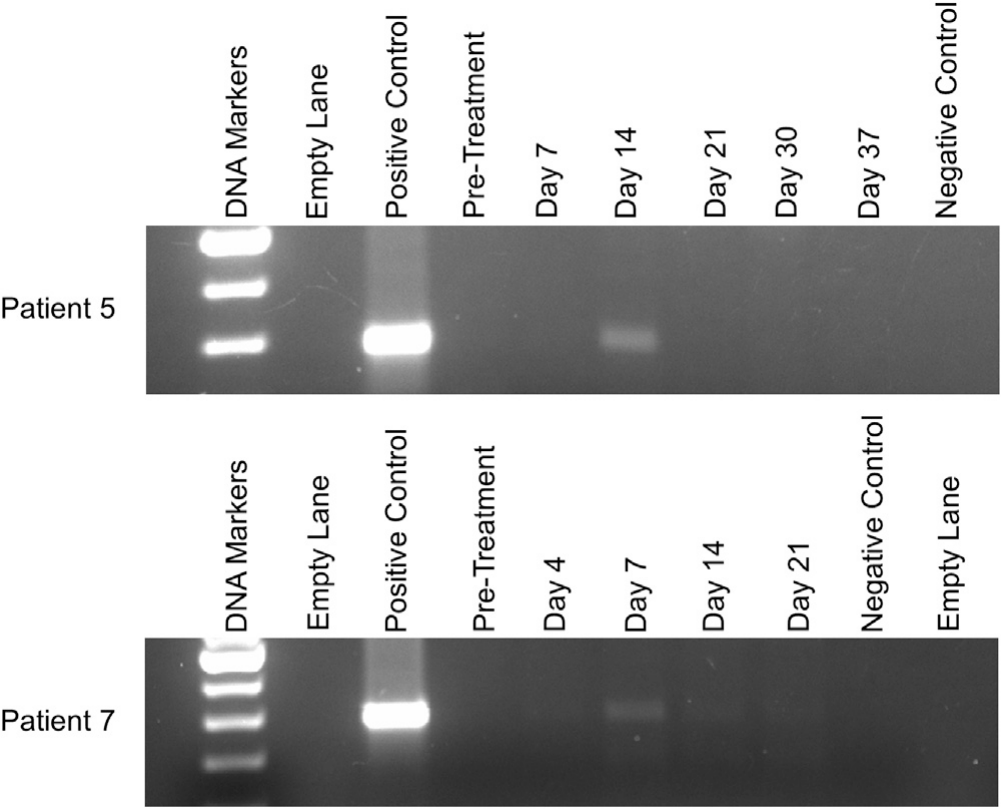
PCR of Ad5-yCD/*mut*TK_SR39_*rep*-hIL-12 DNA in blood Positive control consisted of purified Ad5-yCD/*mut*TK_SR39_*rep*-hIL-12 DNA (1 × 10^4^ vp) added to blood from a healthy volunteer. Pretreatment blood was drawn within 30 days of adenovirus injection. The day of each blood draw is indicated above the indicated lanes. Negative control consisted of blood from a healthy volunteer with no Ad5-yCD/*mut*TK_SR39_*rep*-hIL-12 DNA added. PCR data from patients 5 and 7 are shown in the top and bottom panels, respectively.

In previous clinical trials, we have measured indirectly the persistence of adenovirus DNA following intra-tumor injection by conducting PCR on the subject’s blood. Persistence of adenovirus DNA in blood more than a week after injection is evidence that the adenovirus is replicating within the tumor where it is injected. The template of the PCR primers is a sequence common to all of our related oncolytic adenovirus constructs and found specifically in Ad5-yCD/*mut*TK_SR39_*rep*-hIL-12.^9^ Post-injection day 14, Ad5-yCD/*mut*TK_SR39_*rep*-hIL-12 DNA was not detectable in any of the patients who received the lowest dose (1 × 10^11^ vp) and was detectable in only one subject (patient 5) who received 3 × 10^11^ vp (Figure 1).

It is of interest that adenovirus DNA was detectable in the one patient from the second cohort who happened to be the longest survivor in the first two cohorts (10.5 months post adenovirus injection). We also detected Ad5-yCD/*mut*TK_SR39_*rep*-hIL-12 adenovirus DNA on day 7 in only one patient of the highest adenovirus dose cohort (patient 7). This is the patient who had the SAE described above and survived 15.2 months past the adenovirus injection.

### Immune response

**Figure 2:**
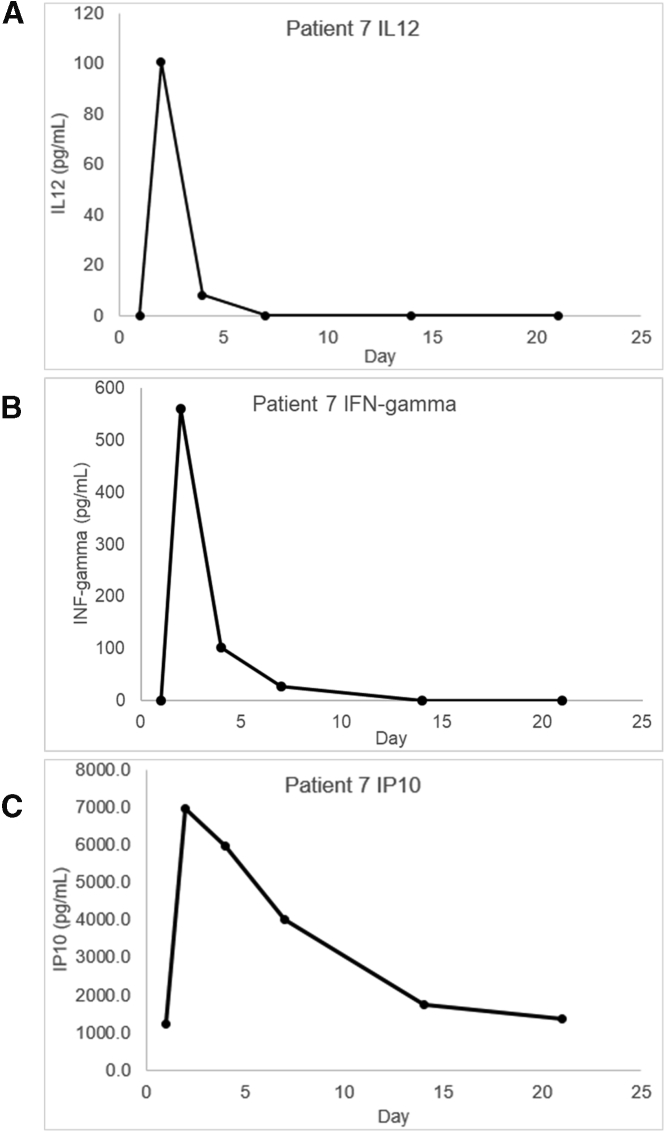
Serum *IL12*gamma, IFNG, and CXCL10 levels in patient 7 Blood was drawn on the day of adenovirus injection prior to the procedure (day 1) and then on the indicated days (2, 4, 7, 14, and 21). Cytokine measurements were made by ELISA using a standard curve specific for each cytokine (0–1,000 pg/mL). Panels A and B show serum IL12 and IFNG, respectively. For CXCL10, the patient samples were diluted 1:100, panel C. Concentration of each cytokine is listed in pg/mL.

To assess immune system activation, we measured serum *IL12*, IFNG, and CXCL10 levels from blood post-adenovirus injection relative to pre-adenovirus injection. In addition, *IL12* expression leads to IFNG production mainly by NK and natural killer T (NKT) cells and activated CD4^+^ T cells and CD8^+^ cytotoxic T cells.^18^ CXCL10 is secreted by monocytes, fibroblasts, and endothelial cells in response to IFNG and possesses anti-angiogenic properties.^19^^,^^20^ Blood was drawn within 30 days prior to adenovirus injection (day 1) and then again on prescribed days after adenovirus injection. Serum *IL12* was not detected in the patients of the first cohort (Table 2). In two of the second cohort patients, relatively low levels of *IL12* (1.1 and 11.3 pg/mL) were detected on day 21, but none of the other time points. In the last cohort, serum *IL12* was detected at relatively high levels (2.9–100.5 pg/mL) on at least day 2 in three of six patients who received the highest dose (1 × 10^12^ vp). The serum *IL12* through day 21 of the best responding patient is shown in Figure 2A.

**Table 2:** Peak serum cytokine day and concentrations

Cohort no.	Patient no.	*IL12*		INFG		CXCL10	
		Day	pg/mL	Day	pg/mL	Day	pg/mL
1	1	not detected		14	88.1	2	672.9
	2	not detected		3	41.8	3	899.3
	3	not detected		not detected		not detected	
2	4	21	1.1	14	51.6	2	1,203.8
	5	21	11.3	not detected		2	1,061.3
	6	not detected		not detected		2	447.1
3	7	2	100.5	2	561.0	2	6,971.9
	8	2	2.9	2	45.5	2	2,564.6
	9	2	23.1	2	193.3	2	3,830.1
	10	not detected		21	75.6	2	1,302.8
	11	not detected		3	91.1	3	5,690.1
	12	not detected		2	165.7	2	5,239.1

Serum IFNG was detected in 9 of 12 subjects, including two of three in cohort 1, one of three in cohort 2, and all six patients of cohort 3 (Table 2). In most of the patients, the peak detected serum IFNG was detected at the earliest time point (day 2 or 3). Subjects 1 and 4 had peak IFN-γ on day 14, and patient 10 had elevated IFNG on day 21. Peak serum IFNG concentrations (relative to pretreatment levels) ranged from 41.8 to 561.0 pg/mL. As with *IL12*, the highest serum concentration of IFNG was observed in patient 7 (Figure 2B). In all patients, serum CXCL10 peaked at earliest time point and then dropped in subsequent measurements. Interestingly, the concentration of CXCL10 at the earliest time point after adenovirus injection appears to be related to the adenovirus dose. The average CXCL10 concentrations of cohorts 1, 2, and 3 were 786.1 (range 672.9–899.3), 904.1 (447.1–1,203.8), and 4,266.4 (1,302.8–6,971.9) pg/mL, respectively (Table 2). Serum CXCL10 was highest (6,971.9 pg/mL) and persisted the longest (detected on days 2, 4, 7, 14, and 21) in patient 7 (Figure 2C).

### Serum NK/CD4^+^/CD8^+^ cell populations

**Figure 3:**
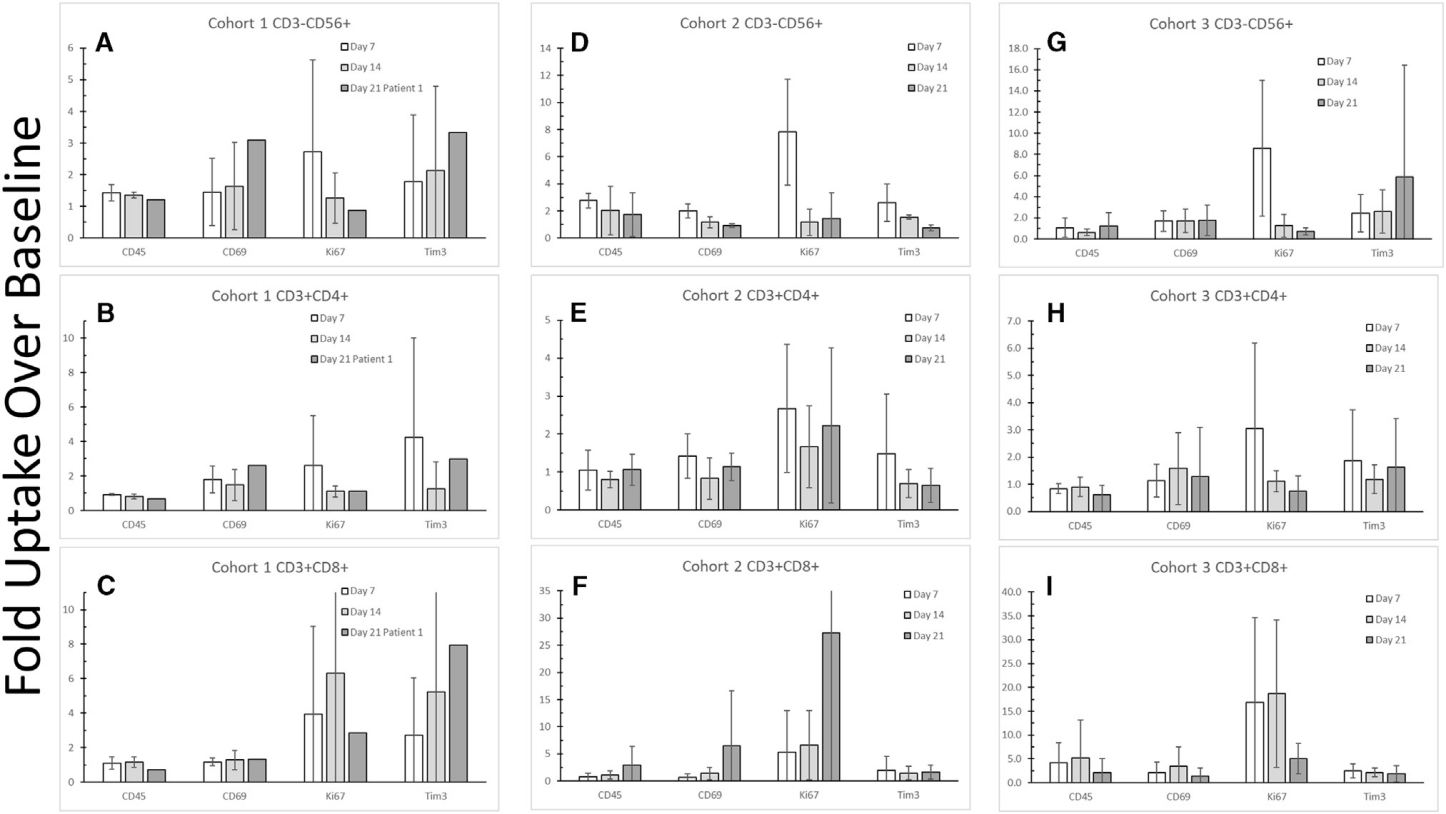
Peripheral blood mononuclear cell (PBMC) counts and markers Blood was drawn pre- and post-adenovirus injection (day 1). PBMCs were harvested and frozen from each patient. Cells were thawed; stained with antibodies against CD3, CD56, CD4, CD8, CD69, CD45(RO), Ki67, and Tim3; and subjected to flow cytometry. All data are reported as group mean fold-increase over baseline blood collected prior to adenovirus injection, which occurred on day 1. Error bars show standard deviation. Day 21 data for cohort 1 were limited to patient 1 only. Samples from cohort 1 are shown in panels A-C; cohort 2 in panels D-F; and cohort 3 in panels G-I.

Circulating PBMCs were analyzed by flow cytometry as a measure of immune system activation. Except for one patient, blood was drawn once prior to adenovirus injection (baseline) and then on indicated days post adenovirus injection (adenovirus injection on day 1). There was no preinjection blood draw obtained for patient 2 (cohort 1). Frozen PBMCs from each patient were thawed, stained with antibody, and analyzed by flow cytometry. Specific antibodies to CD3, CD56, CD4, and CD8 were used to segregate and count immune cell populations (e.g., NK, T helper, and T cytolytic cells). Antibodies were used against CD45(RO), CD69, Ki67 and Tim3. The first three markers indicate that the immune system was activated following treatment. Tim3 is an immune checkpoint protein and indicates that the immune system was supressed following treatment.^21^, ^22^, ^23^ CD45RO is a maturation marker, whereas CD69 and Ki67 indicate immune cell activation and proliferation, respectively. The data were reported as fold increase over the pretreatment blood draw for each cohort. More cells found in subjects’ blood after adenovirus injection than before were positive for markers indicating matured, activated, or proliferating immune cells. For example, on day 7 post adenovirus injection, there was a nearly 3-fold increase in the number of CD3^−^CD56^+^ NK cells expressing the proliferation marker Ki67 in cohort 1 (Figure 3A). In cohorts 2 and 3, there were 8-fold increases in the number of NK cells expressing Ki67 on day 7, respectively (Figures 3D and 3G).

Increased numbers of cells expressing Ki67 were observed also in CD3^+^CD4^+^ T helper cells (Figures 3B, 3E, and 3H); in cohort 2, the level of CD3^+^CD4^+^ cells expressing Ki67 persisted through day 21 (Figure 3E). Ki67 expression in CD3^+^CD8^+^ T cells also increased over pretreatment levels (Figures 3C, 3F, and 3I). These changes in Ki67 expression indicate that the immune system was activated following adenovirus injection into the pancreatic tumors. Another indication in the activation of the immune system was observed in the increase in the number of cells that expressed CD69, which following immune system activation is one of the earliest inducible lymphoid cell surface glycoproteins.^22^ The increases in the counts of cells expressing CD69 that was observed in many of the cell populations did not appear to be dependent upon the dose of adenovirus (Figure 3). CD45RO expression is associated with activated and CD3^+^CD8^+^ T cytolytic memory cells. Of note, we observed in cohort 3 that CD3^+^CD8^+^ T cells showed an initial increase in the number of cells expressing CD45RO, but then by day 21, the number of cells returned nearly to baseline (Figure 3I). This observation is consistent with the “On-Off-On” model for memory T cells.^26^ In this model, T cells are activated when the T cell receptor binds to an antigen, causing the cell to turn “On” and proliferate. After the antigen is cleared, these cells are turned “Off,” and some circulate through the body as T memory cells. When the specified antigen is encountered again, these cells are turned “On” and mount an effective response after reactivation. Finally, it is of interest that the number of CD3^+^CD8^+^ T cells expressing the immune checkpoint, Tim3, increased over time relative to pretreatment levels in cohort 1 (Figure 3C). In contrast, the number of CD3^+^CD8^+^ T cells in cohorts 2 and 3 expressing Tim3 largely stays at near pretreatment levels or even decreased over time (Figures 3F and 3I). Tim3 plays a role in CD3^+^CD8^+^ T cell exhaustion.^23^, ^24^, ^25^

## Discussion

The primary endpoint of the study was to determine the tolerability and MTD of oncolytic adenovirus-mediated suicide and *IL12* gene therapy and prodrugs in metastatic pancreatic cancer patients. Ad5-yCD/*mut*TK_SR39_*rep*-hIL-12 doses were escalated from 1 × 10^11^ to 1 × 10^12^ vp in three half-log steps followed by 7 days of 5-FC prodrug therapy starting on day 2 and then chemotherapy starting 21 days after adenovirus injection. Like other clinical trials using similar oncolytic adenovirus agents, over 90% of the AEs observed in this trial were grade 1 or 2 and required no medical intervention to resolve. In the present study, an SAE possibly related to the study agent was encountered in one subject of the third cohort. Consequently, the third cohort was expanded to include six patients according to protocol. No other SAEs were encountered, hence the MTD was not reached. The study demonstrates that intra-pancreatic administration of Ad5-yCD/*mut*TK_SR39_*rep*-hIL-12 followed by 5-FC prodrug therapy and chemotherapy is safe.

We have completed five phase I clinical trials and one prospective, randomized, controlled phase I/II clinical trial using three similar oncolytic adenoviruses in newly diagnosed and locally recurrent prostate cancer.^9^, ^10^, ^11^, ^12^, ^13^, ^14^ Colleagues have completed a phase I clinical trial in locally advanced pancreatic cancer patients using a similar adenovirus construct, Ad5-yCD/*mut*TK_SR39_*rep*-ADP. They reported that the combination of the oncolytic adenovirus-mediated gene therapy combined with gemcitabine was well tolerated in this population of patients.^27^ The median progression-free survival was reported to be 11.4 months. In addition, we have recently completed a phase I trial using Ad5-yCD/*mut*TK_SR39_*rep*-hIL-12 in locally recurrent prostate cancer patients (unpublished data: K Barton, F Siddiqui, S Freytag, and B Movsas). Although there are many similarities with the current clinical trial and the others mentioned, the present study is the first to include the following: (1) use of *IL12* immuno-gene therapy in combination with our oncolytic adenovirus suicide gene therapy, and (2) use of our therapeutic approach in metastatic (pancreatic) disease.

*IL12* therapy (including *IL12* gene therapy) has been employed in several phase I clinical studies for cancer with mixed results.^28^ The underlying interest in *IL12*-mediated tumor immunotherapy is based upon its well-characterized ability to activate both the innate and adaptive arms of the immune system. Under ideal situations, *IL12* expression by professional antigen-presenting cells acts on NK, CD4^+^, and CD8^+^ cells, causing them to produce IFNG and eventually CXCL10, both of which have many antitumor activities and promote coordinated antitumor immunity.^19^^,^^20^ In prior clinical applications, however, *IL12*-mediated tumor immunotherapy has not demonstrated significant efficacy and instead, there were cases of unacceptable toxicities with this approach. Additionally, severe side effects, including deaths of study patients, often have been associated with systemic administration of *IL12*. Targeted or tumor-specific expression of *IL12* appears to provide the benefits of *IL12*-mediated tumor immunotherapy with less of the toxicity observed with systemic administration.^18^ This is the approach we employed by engineering our oncolytic adenovirus that expresses the suicide fusion genes yCD/*mut*TK_SR39_ and Ad5-yCD/*mut*TK_SR39_*rep*-hIL-12 to also express the hIL-12 gene.^16^ One of the benefits of using an oncolytic adenovirus is its ability to convert “immunologically cold” tumors into “immunologically hot” tumors. Cold tumors are characterized by having low levels of immune cell populations, whereas immune cells are present in hot tumors.

Others have argued that low mutational burden is of equal importance as lack of immune cells in the definition of “immunologically cold” tumors.^29^ Mutation burden is the property of the tumor genome that results in novel DNA sequences leading to novel polypeptide sequences presented on tumor cells. These novel polypeptides act as neoantigens for the immune system to recognize and target tumor cells for destruction. The combination of these two characteristics (absence of immune cells and low mutation burden) in a tumor limits the ability of the immune system to act on a tumor. Pancreatic tumors are characterized as cold tumors because there is often an absence of immune cells in the tumor and the low mutation burden. Our approach of oncolytic adenovirus-mediated suicide and *IL12* gene therapy in combination with chemotherapy could attract immune cells into the tumor, thereby reversing the normal situation found in pancreatic cancers (i.e., absence of immune cells). It is likely that the immune cells attracted to the tumor following Ad5-yCD/*mut*TK_SR39_*rep*-hIL-12 treatment are actually targeting the adenovirus, or more likely the cells infected with the adenovirus. We also observed in these patients the temporary increases in serum cytokines (such as *IL12*, IFNG, and CXCL10) in response to adenovirus injection. Likewise, following Ad5-yCD/*mut*TK_SR39_*rep*-hIL-12 injection, we observed in many patients increased PBMCs and a subset of proliferation markers on those cells. At 12 months post-adenovirus injection, two of six patients who received 1 × 10^12^ vp (highest dose) of Ad5-yCD/*mut*TK_SR39_*rep*-hIL-12 and completed the 5-FC prodrug therapy and several rounds of chemotherapy have stable disease, not only in the treated tumors but also the metastatic lesions found in liver and lung. Patients 7 and 8 received gemcitabine or gemcitabine/protein-bound paclitaxel (Abraxane), respectively, after adenovirus and 5-FC therapies. The progression-free survivals of patients 7 and 8 were 11.5 and 16.1 months, compared with a median expected duration of 3.3 months for gemcitabine-treated patients. Likewise, their overall survival durations were 15.2 and 20.0 months compared with an expected 6.4 months for gemcitabine alone. No conclusions can be made from these data, but hypotheses regarding the current situation of their disease can be formulated (see below).

A second novel aspect of this study is that metastatic pancreatic patients were treated with the oncolytic adenovirus-mediated suicide and *IL12* gene therapy. In previous studies, patients with newly diagnosed or locally recurrent disease were enrolled. In fact, evidence of disseminated disease was an exclusion criterion in our previous trials. All patients in this trial, by contrast, had disseminated disease. This presented both a potential hazard and an opportunity. The hazard would be that in a study where one tumor was treated and other tumors were not, then the untreated tumors may progress and obscure both the toxicity and efficacy data of the study agent that could have been obtained. The opportunity would be realized if the study agent would result in the development of antitumor immunity, which would lead to effects in the disseminated tumors found in the liver and lungs. It appears that the latter case may have happened in the patients in cohort 3. All surviving patients have had at least one follow-up CT assessment to evaluate tumor response. The median follow-up for CT assessments is 16.0 months. In two cases, the primary or treated tumors and metastatic tumors (untreated) were stable. The median progression-free survival for cohort 3 is 10.6 (range, 2.2–17.4) months. For comparison, the median progression-free survival time of metastatic pancreatic cancer patients who receive FOLFIRINOX or gemcitabine alone was 6.4 or 3.3 months, respectively.^5^

Although encouraging signs of efficacy were observed in the subjects who received the highest dose of Ad5-yCD/*mut*TK_SR39_*rep*-hIL-12, no conclusions regarding efficacy can be drawn from a phase I study. However, hypotheses can be generated, which then can be the basis for future clinical studies. For example, because the MTD was not reached, it is uncertain whether Ad5-yCD/*mut*TK_SR39_*rep*-hIL-12 can be administered at higher doses (e.g., 3 × 10^12^ vp). The efficacy data reported here indicate that dose escalation may be warranted. However, rather than conduct a simple phase I/II dose-escalation trial to determine the MTD, conducting a continuous reassessment method (CRM) phase I/II study might be a better use of resources and effort. The CRM would facilitate potentially a rapid determination of the MTD through the phase I dose escalation to maximize the number of patients treated at or near the MTD for the phase II component of the trial. The study design of this proposed study would be like the current study in that chemotherapy would be combined with the oncolytic adenovirus-mediated suicide and *IL12* gene therapy in patients with metastatic pancreatic cancer.

Pancreas tumors are characterized by having variable levels of interstitial pressure and fibrosis secondary to a dense tumor stroma. These characteristics when present at high levels may limit the effectiveness of injecting adenovirus into pancreatic tumors as a result of leakage from the injection site or reduced adenovirus absorption or transduction of the tumor itself. The variable nature of the pancreatic tumor microenvironment may partially explain why some patients in the higher dose cohort responded worse than others. A method to assess or image the quality of the adenovirus injection may be helpful in future clinical studies. Ad5-yCD/*mut*TK_SR39_*rep*-hIL-12 expresses *mut*TKSR39, the activity of which can be imaged non-invasively using positron emission tomography (PET) using a thymidine kinase substrate, 9-(4-[^18^F] fluoro-3-hydroxymethylbutyl guanine) ([^18^F]-FHBG). Following intravenous injection of [^18^F]-FHBG, the thymidine kinase substrate would be concentrated in cells infected with Ad5-yCD/*mut*TK_SR39_*rep*-hIL-12 and could be imaged non-invasively using PET. PET imaging is quantitative and would allow us to assess the efficacy of Ad5-yCD/*mut*TK_SR39_*rep*-hIL-12 injections into the pancreas. We have demonstrated the feasibility of imaging an adenovirus expressing thymidine kinase injected into the pancreas of a canine preclinical model.^30^

Regardless of the next clinical study using Ad5-yCD/*mut*TK_SR39_*rep*-hIL-12, the experiences of this clinical study should inform the design of future studies. For example, hypotension has been noted as an adverse effect of *IL12* plasmid or gene therapy.^31^ High vigilance of AEs such as hypotension related to *IL12* therapy is recommended, including a suggestion to clinicians to urge caution with patients who are on hypertension medicine while receiving Ad5-yCD/*mut*TK_SR39_*rep*-hIL-12. Other AEs associated with *IL12* therapy include flu-like symptoms and hematological complications, such as neutropenia and thrombocytopenia. One of the known side effects of suicide gene therapy in our experience is depression of blood cell counts (i.e., neutropenia and thrombocytopenia).^18^ Hence there is a possible combined effect of *IL12* and the suicide genes on depressing blood cell counts. The administration of oncolytic adenovirus constructs can result in flu-like symptoms, which can be exacerbated by *IL12*. The designers of a future dose-escalation study involving suicide and *IL12* gene therapy should be aware of these potential complications. Considering the one SAE possibly related to the study agent, the safety profile of Ad5-yCD/*mut*TK_SR39_*rep*-hIL-12 is considered acceptable and similar in safety profile to the three other oncolytic adenovirus constructs that we have assessed in clinical studies.

## Materials and methods

### Study design

This was an IRB-approved phase I dose-escalation study designed to evaluate the safety of the replication-competent adenovirus, Ad5-yCD/*mut*TK_SR39_*rep*-hIL-12. The patient population consisted of metastatic pancreatic adenocarcinoma patients. Primary endpoints included determining the MTD and DLTs of the study agent up to day 21 post adenovirus injection. The secondary endpoint included rates of grade 3 AEs using the National Cancer Institute’s (NCI’s) Common Terminology Criteria for AEs version 4.03 (CTCAE v.4.03). Exploratory endpoints included possible association between primary/secondary outcomes and the following: (1) serum hIL-12, IFNG, and CXCL10 levels; (2) circulating CD3^−^CD56^+^ NK, CD3^+^CD4^+^ T helper cells, or CD3^+^CD8^+^ T cells; and (3) expression of a subset of proliferation markers (e.g., CD69, CD56, and Ki67) and a checkpoint marker (Tim3) on the serum NK and T cell populations. An independent DSMB supervised all aspects of the study, including the collection and review of all toxicity data.

There were three doses of adenovirus injected in this trial (1 × 10^11^, 3 × 10^11^, and 1 × 10^12^ vp). Each member of the three patient cohorts received a single dose of adenovirus injected directly into the pancreatic tumors (details below). In the event of a DLT (defined below), the cohort would be expanded from three to six patients. If a second DLT would be observed (DLT observed in 33% of the patients of the expanded cohort), then the next lower dose cohort would be expanded to six patients. If the ≥33% of the patients in the first cohort had developed a DLT, then the trial would have been terminated.

A DLT was defined as any toxicity (possibly, probably, or definitely) related to the investigational agent (Ad5-yCD/*mut*TK_SR39_*rep*-hIL-12) that results in any of the following outcomes: grade ≥3 allergic reaction or generalized urticaria, grade ≥3 cardiovascular or neurological toxicity, and grade ≥3 hematologic or non-hematologic toxicity of any duration (except for grade 3/4 lymphopenia recovered within 2 weeks; grade 3/4 anemia, leukopenia, neutropenia, or flu-like symptoms that are reversible within 72 h; grade 3 SGOT/SGPT serum levels that are reversible with 72 h; grade 3/4 hyperglycemia that can be explained by patient’s baseline comorbidity [e.g., diabetes]; and grade 3/4 electrolyte imbalances that are reversible within 72 h).

Eligibility criteria included histologically proven (biopsy or cytology) metastatic pancreatic adenocarcinoma in patients ≥18 years old. No prior treatment, including surgery, chemotherapy, radiotherapy, or biological therapy, for pancreatic cancer was allowed. Zubrod performance score of 0–2 within 30 days of registration was required. Adequate baseline organ functions were required within 30 days of registration. Laboratory values required for enrollment included serum creatinine ≤1.8 mg/dL or creatinine clearance >50 mL/min/m^2^, platelet count >100,000/μL, absolute neutrophil count >1,000/μL, hemoglobin >8.0 g/dL, bilirubin >2.0 mg/dL, and AST/SGOT and ALT/SGPT <3.0 times upper limit of normal (ULN). The AST/SGOT and ALT/SGPT requirements were relaxed to <5.0 times ULN for patients with liver metastases.

Women of childbearing age and male participants agreed to use medically effective means of birth control throughout and for 60 days beyond the treatment phase of the study. Patients on anticoagulation therapies were monitored for signs or symptoms of bleeding. Subjects were required to possess the ability to provide informed consent and express a willingness to meet all expected requirements for the protocol for the duration of the study.

Exclusion criteria included any of the following: pregnant or lactating women, clinical or laboratory evidence of pancreatitis based upon the discretion of the treating physician, a serious non-malignant disease (e.g., congestive heart failure or uncontrolled infections) that at the discretion of the treating physician would compromise study objectives, and major surgery within 3 months of study registration. Patients presenting with non-adenocarcinoma of the pancreas (e.g., islet cell tumor, such as neuroendocrine tumors, cystic neoplasms, or peri-ampullary carcinoma) were excluded. The presence of an acute infection defined as any viral, bacterial, or fungal infection that required specific therapy within 72 h of study initiation, previous history of liver disease (including hepatitis), positive serological tests for hepatitis B or C at baseline, immunosuppressive therapy including systemic corticosteroids, impaired immunity or susceptibility to serious virus infections, an allergy to any product used in this study, or serious medical or psychiatric illness or concomitant medication that might interfere with the subject’s ability to tolerate or complete the study were also exclusionary criteria. The trial was registered on the NIH National Library of Medicine database (https://www.clinicaltrials.gov/) with the NTC registration code ClinicalTriagls.gov: NCT03281382.

### Design and manufacturing of Ad5-yCD/*mut*TK_SR39_*rep*-hIL-12 adenovirus

The design of Ad5-yCD/*mut*TK_SR39_*rep*-hIL-12 adenovirus was constructed using techniques such as those used previously.^16^ In brief, the left-end vector containing the yCD/*mut*TK_SR39_ fusion gene cassette under transcriptional control of a human cytomegalovirus (CMV) promoter has been described previously.^8^ The source for hIL-12 was pUNO1-IL-12 (p40::p35) (InvivoGen, San Diego, CA, USA). The 1,611-bp encoding sequence was amplified by PCR and cloned between a CMV promoter and SV40 polyadenylation sites of pCA14 (Microbix, Mississauga, ON, Canada). The CMV-hIL-12-SV40 expression cassette was cloned into pBGH8k at the *SwaI* and *PacI* endonuclease sites. The adenovirus was generated by calcium phosphate precipitation of the pCA14-yCD/*mut*TK_SR39_ and pBGH8k-hIL-12 plasmids linearized with *PvuI* and *ClaI*, respectively. The precipitated DNA was co-transfected in AD293 cells and then purified (Adenopure; Puresyn, Malvern, PA, USA). DNA sequencing was used to verify sequences of the suicide genes and hIL-12. The cytopathic properties of the Ad5-yCD/*mut*TK_SR39_*rep*-hIL-12 adenovirus were tested in several different cell lines. Clinical-grade (Good Manufacturing Process [GMP]) Ad5-yCD/*mut*TK_SR39_*rep*-hIL-12 adenovirus was made at the Baylor College of Medicine Gene Vector Laboratory (Houston, TX, USA). A Master Viral Bank was generated and tested for safety, sterility, titer, endotoxin, identity (PCR and DNA sequencing), transgene expression, and potency, as described by Freytag et al.^9^ All safety tests were negative. All identity and potency tests were similar to the laboratory-grade stock adenovirus. The stock concentration of the adenovirus was 1.0 × 10^12^ vp/mL and supplied in a clear, frozen liquid in individual vials of 1.1 mL. Each vial was labeled serially. One vial would be thawed, and the appropriate amount of adenovirus stock solution would be diluted for each patient (see details below).

### Treatment

Informed consent was obtained prior to study-related procedures being performed. Under conscious sedation (combination of meperidine and midazolam or propofol), a linear echoendoscope was inserted into the patient’s stomach or duodenum under direct visualization. The dimensions of the pancreas and the adenocarcinoma mass were acquired using the echoendoscope. The study agent (Ad5-yCD/*mut*TK_SR39_*rep*-hIL-12 + phosphate-buffered saline [PBS]) would be prepared so that the total injection volume was 10% of the tumor mass. Under direct ultrasonographic visualization, a 22G Wilson-Cooke Echotip needle was inserted into the tumor through the safest approach (trans-gastric or -duodenal) as determined by the interventional gastroenterologist. The gastric approach was the preferential choice to reduce the risk for duodenal perforation. The needle tip was placed at the distal edge of the tumor (relative to needle entry site), and the study agent was injected along the tract as the needle was withdrawn. Multiple needle passes were required for larger tumors to provide good coverage. Forty-eight hours postprocedure, patients received a 7-day course of 5-FC (150 mg/kg/day, 4 times a day orally; Ancobon). Patient compliance was monitored by the study nurse. Twenty-one days after adenovirus injection, patients began to receive standard-of-care systemic therapy at the discretion of the treating physician. The preferred therapies were FOLFIRINOX (5-FU, leucovorin, irinotecan, oxaliplatin) or Gemzar/Abraxane (gemcitabine + albumin-bound paclitaxel). Cycles of the preferred therapies were continued until the patient’s death or at the discretion of the treating oncologist when considering toxicities resulting from the chemotherapy regimen. Other/secondary options for chemotherapy would be implemented as determined by the treating physician.

### Assessments

Toxicity assessments were performed once a week prior to the start of chemotherapy (first 3 weeks) and then at scheduled follow-up visits at 3, 6, 9, 12, 18, and 24 months. Toxicities were graded using the CTCAE v.4.03. Case report forms (CRFs) specifically designed for this trial were used to document AEs.

Evaluations prior to the start of chemotherapy (day 21) included complete weekly blood counts; blood chemistries; comprehensive metabolic panel; presence of Ad5-yCD/*mut*TK_SR39_*rep*-hIL-12 viral DNA and infectious adenovirus in blood, serum *IL12*, IFNG, and CXCL10; and physician assessment. Evaluations during chemotherapy were monitored according to standard of care for patients receiving chemotherapy or as clinically indicated and included complete blood counts; blood chemistries; presence of Ad5-yCD/*mut*TK_SR39_*rep*-hIL-12 viral DNA and infectious adenovirus in blood, serum *IL12*, IFNG, and CXCL10; CA19-9 according to standard of care for subjects receiving chemotherapy or as clinically indicated; and provider assessments. Chemotherapy clinical follow-up assessments occurred according to standard of care for metastatic pancreatic cancer patients receiving chemotherapy or as clinically indicated. These evaluations included patient history; physical examinations; performance status; complete blood counts; blood chemistries; presence of Ad5-yCD/*mut*TK_SR39_*rep*-hIL-12 viral DNA, serum *IL12*, IFNG, and CXCL10; CA19-9; CT scans (pancreas and chest protocols every 2 months); and toxicity assessments at every scheduled follow-up visit using CRFs designed for this trial. Notably, the presence of Ad5-yCD/*mut*TK_SR39_*rep*-hIL-12 viral DNA in blood, serum *IL12*, IFNG, and CXCL10 levels were monitored at every blood draw until not detected in two consecutive measurements.

### Administration of 5-FC

5-FC (Ancobon; Roche Laboratories, Basel, Switzerland) was administered orally beginning day 3 post injection and continued for 7 consecutive days. A total of 150 mg/kg/day was given.

### PCR of Ad5-yCD/*mut*TK_SR39_*rep*-hIL-12 adenoviral DNA in blood

The assessment of adenovirus DNA in subjects’ blood has been described previously.^9^ In brief, blood was obtained before the adenovirus was injected to obtain a baseline value for each patient. Then blood was drawn at prescribed time points following adenovirus injection to assess the level of serum Ad5-yCD/*mut*TK_SR39_*rep*-hIL-12 adenovirus DNA. The PCR primers hybridize the linker located between the yCD and the mutant HSV-1 TK gene. The PCR product is 388 bases in length and specific for the yCD/*mut*TK_SR39_ fusion gene. To generate a standard curve, human volunteer blood was spiked with Ad5-yCD/*mut*TK_SR39_*rep*-hIL-12 adenovirus DNA from 50 vp/mL to 5.0 × 10^9^ vp/mL serial dilutions. The sensitivity of the assay is 50 vp/mL.

### Flow cytometry

Blood was drawn once pretreatment (days −30 to −1) and then again on days +3, +7, +14, and +21 to isolate PBMCs, which were stored in liquid nitrogen until analysis. The PBMC freezing media consisted of 10% dimethylsulfoxide (DMSO) and 90% heat-inactivated fetal bovine serum. PBMCs were quickly thawed in a 37°C H_2_O bath, then washed once in room temperature 1X PBS and centrifuged at 1,500 rpm for 10 min at 4°C. Supernatants were removed, and contaminating red cells were lysed, while the remaining mononuclear cells were fixed at room temperature, for 10 min, in FACSLyse solution (BD Biosciences, San Jose, CA, USA). Cells were pelleted by centrifugation at 1,500 rpm at 4°C for 10 min. After supernatant removal, the cells were resuspended in wash buffer (1X PBS, 5% bovine serum albumin, 10% sodium azide), then aliquoted to labeled wells of a Falcon 96-well round-bottom tissue culture plate (Corning, Durham, NC, USA). Plated cells were centrifuged at 1,500 rpm for 5 min at 4°C; then the supernatants were removed without dislodging the pellets. Cells were blocked by incubation in FC block (BioLegend, San Diego, CA, USA) at room temperature for 10 min. Following pre-blocking, plated cells were centrifuged, as above, and the cells were incubated in the dark at room temperature for 30 min with a cocktail of antibodies to surface antigens containing anti-human CD3 conjugated with allophycocyanin-Cy7 (APC-Cy7), anti-human CD56 conjugated with PEDazzle 594, anti-human CD45RO conjugated with R-phycoerythrin-Cy7 (PE-Cy7), anti-human CD8 conjugated with BV510, anti-human CD69 conjugated with PE, anti-human Tim3 conjugated with BV421 (BioLegend), and anti-human CD4 conjugated with peridinin chlorophyll protein Cy5.5 (PerCP-Cy5.5,BD Biosciences). Following incubation, the plated cells were washed in washing buffer and then centrifuged, as detailed above, and the supernatant was removed with this process being repeated for an additional cycle. Afterward, cells were incubated in 1X Fix/Perm buffer (eBioscience, Waltham, MA, USA) in a 4°C refrigerator for 45 min in the dark. Cells were pelleted by centrifugation and the pellet resuspended in 1X Perm buffer (eBioscience) and centrifuged, as described above. Cells were then incubated with 2% normal goat serum (BioLegend), diluted with 1X Perm buffer (eBioscience), for 20 min on ice in the dark. Afterward, pellets were incubated with anti-human Ki67 conjugated with APC (BioLegend) or mouse IgG1 k isotype control conjugated with APC (BioLegend) on ice in the dark for 30 min. After incubation, cells were washed twice with 1X Perm buffer (eBioscience) and then resuspended in wash buffer and transferred to Falcon 12X75 5-mL round-bottom polystyrene tubes (Corning) for flow cytometry. Cells were acquired using a four-laser (BD Biosciences) LSRFortessa (BD), and 100,000 gated events were collected for each sample. Analysis of results was performed using BD FACSDiva software V.8.0.1 (BD Biosciences). Cells were identified using their light scattering properties in the forward and side scatter channels, and gates were positioned using single stain, fluorescence minus one (FMO), and isotype controls.

## Supplemental information

Table S1. Adverse event by system organ class
